# A Fast Prediction Model for Liquid Metal Transfer Modes during the Wire Arc Additive Manufacturing Process

**DOI:** 10.3390/ma16072911

**Published:** 2023-04-06

**Authors:** Jiaqi Ouyang, Mingjian Li, Yanping Lian, Siyi Peng, Changmeng Liu

**Affiliations:** 1 Institute of Advanced Structure Technology, Beijing Institute of Technology, Beijing 100081, China; 2 School of Mechanical Engineering, Beijing Institute of Technology, Beijing 100081, China

**Keywords:** metal transfer, liquid bridge, prediction model, process parameter window, additive manufacturing

## Abstract

The liquid metal transfer mode in wire arc additive manufacturing (WAAM), plays an important role in determining the build quality. In this study, a fast prediction model based on the Young–Laplace equation, momentum equation, and energy conservation, is proposed, to identify the metal transfer modes, including droplet, liquid bridge, and wire stubbing, for a given combination of process parameters. To close the proposed model, high-fidelity numerical simulations are applied, to obtain the necessary inputs required by the former. The proposed model’s accuracy and effectiveness are validated by using experimental data and high-fidelity simulation results. It is proved that the model can effectively predict the transition from liquid bridge, to droplet and wire stubbing modes. In addition, its errors in dripping frequency and liquid bridge height range from 6% to 18%. Moreover, the process parameter windows about transitions of liquid transfer modes have been established based on the model, considering wire feed speed, travel speed, heat source power, and material parameters. The proposed model is expected to serve as a powerful tool for the guidance of process parameter optimization, to achieve high-quality builds.

## 1. Introduction

Wire arc additive manufacturing (WAAM) technology has gained increasing attention thanks to its high deposition rate, low equipment and material consumption, and large forming size [1,2]. However, it still suffers from difficulties in controlling morphology, including hump defects, surface roughness, and deviations in shape and size, and difficulties in controlling mechanical properties, such as coarse microstructures caused by high heat input [3,4]. The metal transfer behavior is one of the most important issues affecting the morphology and mechanical properties of parts formed by WAAM. It has been demonstrated that unstable metal transfer can lead to uneven surfaces and internal porous defects in the solidified parts and, therefore, significantly affect the forming quality and mechanical properties [5,6].

In general, three types of metal transfer modes are observed in experiments: the droplet mode, the liquid bridge mode, and the wire stubbing mode [7,8]. Among them, the liquid bridge mode is most favorable [8,9]. At this point, the liquid metal enters the melt pool steadily and forms a liquid bridge connecting the melt pool and the wire. As a result, it leads to a smooth melt pool surface and, therefore, a better forming quality. However, achieving such a stable liquid bridge is challenging in practice. For example, when the distance between the wire and the substrate is large enough, a droplet pattern develops, causing surface ripples. Alternatively, if the distance is too small, a stubbing mode tends to develop, where unmelted wire causes severe disturbance to the melt pool, reducing the forming quality and resulting in metallurgical defects. Other factors that affect liquid metal transfer patterns include the wire feed speed, travel speed, and input power, among others. For controlling the metal transfer pattern, these process parameters must be carefully selected, based on an accurate model, considering multiple physical fields and violent multi-phase flow.

Much work has been devoted to the metal transfer modes in wire feed additive manufacturing processes. Abioye et al. [8] analyzed the influence of heat input, travel speed, and wire feed speed on the deposition cladding layer, and established the process parameter window of energy per unit length of track and wire deposition volume per unit length of track, to predict the liquid bridge transfer and droplet transfer modes. Luo et al. [10] used the arc information in the manufacturing process to identify the droplet transfer mode, and established a quadratic polynomial relationship between droplet transfer frequency and arc power. Scotti et al. [11] addressed the importance of the distance between the wire and substrate in controlling different metal transfer behaviors. Wu et al. [12] reported that when the distance between the electrode and the substrate increases, the liquid bridge transfer mode changes to the droplet transfer mode. Zhao et al. [13] pointed out that the liquid bridge mode had good comprehensive performance, while the wire stubbing mode was unstable and could cause unmelted defects.

To better understand the physical mechanism of heat transfer and fluid flow behavior during metal transfer, many numerical investigations have been conducted. Tang et al. [14] used the level-set method to identify the free surface, and investigated the periodic impact of droplets on the melt pool. Hu et al. [15] investigated the liquid bridge transfer mode, considering the influence of the wire feed. They proposed a dimensionless parameter called “slenderness number”, to roughly estimate whether the liquid bridge can maintain a stable configuration. Bishal et al. [16] used a volume of fluid (VOF) model to study the metal transfer behaviors and presented a corrected formula of droplet detachment in the WAAM process. Chen et al. [17] numerically studied metal transfer modes at different wire feed speeds, and found that the periodic flow pattern of the melt pool, caused by metal transfer impact, leads to ripple and even hump defects, resulting in uneven forming quality. Despite the effectiveness in analyzing metal transfer behaviors, the numerical models above are limited by high computational cost, especially when used in the optimization process. Therefore, a proper model to rapidly predict metal transfer mode, is urgently needed.

This work proposes a prediction model to identify the metal transport mode (droplet, liquid bridge, or wire stubbing) in the WAAM problem. Based on the balance of surface tension and pressure difference, the model can provide the morphology of the liquid bridge and the critical condition for turning into droplet mode. Derived based on energy conservation, the model can also predict whether the liquid bridge mode will turn into a wire stubbing mode. The prediction model requires only a few high-fidelity numerical simulations to calibrate the input parameters, and can accurately predict similar problems for a given combination of process parameters. Validation cases suggest that the prediction model is computationally efficient compared to numerical simulations, with sufficient accuracy, which is very beneficial for optimizing process parameters. Finally, a series of calculations are performed based on the proposed model. A window of process parameters for the WAAM problem is presented, considering wire feed speed, travel speed, heat source power, and material parameters. In contrast to high-fidelity numerical models, the presented approach offers significantly higher efficiency, since it does not need to solve the entire deposition process. Compared to experiments, it can predict liquid metal transfer modes with acceptable accuracy, in an economical and efficient manner. Therefore, the proposed model can serve as a powerful tool for optimizing process parameters when a large number of liquid transfer mode predictions are necessary.

## 2. Prediction Model for Liquid Metal Transfer Modes

### 2.1. Brief Introduction to Metal Transfer Modes

**Figure 1:**
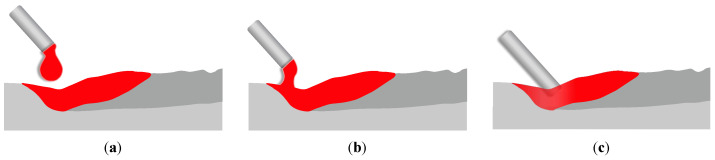
Metal transfer modes in the WAAM process: (**a**) droplet transfer mode, (**b**) liquid bridge mode, and (**c**) wire stubbing transfer mode.

In WAAM, the liquid metal transfer mode plays an important role in the melt track’s morphology and the component’s fabrication quality. The three types of transfer mode, droplet, liquid bridge, and wire stubbing, are illustrated in Figure 1, and briefly introduced as follows. In the droplet transfer mode, the liquid metal periodically enters the melt pool as droplets. The liquid bridge mode denotes that the wire and substrate are bridged by continuous liquid metal. In the wire stubbing mode [18,19,20], the solid wire fails to completely melt before entering the melt pool, leading to severe disturbance to the melt pool. Generally, the liquid bridge mode is most favorable, while the other two modes, especially the wire stubbing mode, need to be avoided. The following section will analyze the liquid bridge mode and determine the critical conditions for its transformation into the droplet and the wire stubbing modes.

### 2.2. Profile of the Liquid Bridge

**Figure 2:**
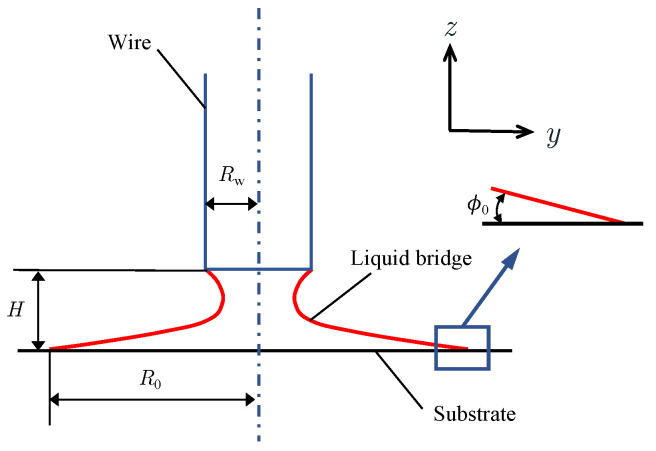
Schematic diagram of the liquid bridge metal transfer mode.

In the liquid bridge mode, the profile of the liquid metal is shown in Figure 2. The shape of the liquid bridge is mainly dominated by surface tension, gravity, and pressure. To describe the continuous liquid bridge, let us first assume that the input power of the arc is sufficient to melt the feed wire into liquid completely. Meanwhile, the shape of the liquid bridge is considered to be axisymmetric. As the Marangoni force acts mainly in the tangential direction, its effect on the liquid bridge profile is exerted indirectly, by influencing the flow velocity, which is ignored here. The pinch effect caused by the magnetic field is not considered. As a result, the surface tension in the normal direction mainly determines the profile. The profile can thus be described by the Young–Laplace equation [21]:

(1) σκ=Δp

where κ is the curvature, σ is the surface tension coefficient, and Δp is the pressure difference across the gas–liquid surface. For a body with cylindrical symmetry, the curvature reads:

(2) $$\kappa =\frac{1}{R{\left(1+R^{\prime 2}\right)}^{1/2}}-\frac{R^{\prime \prime }}{{\left(1+R^{\prime 2}\right)}^{3/2}}$$

where *R* is the radius and superscripts ${}^{\prime }$ and ${}^{\prime \prime }$ denote first- and second-order derivatives, respectively.

To simplify the modeling, the fluid momentum conservation is written as the following formulation, considering the liquid to be stable:

(3) ∇p=ρg

where ρ is the density and g is the gravitational vector. Assuming the pressure at the top of the liquid radius is atmospheric pressure $p_{\mathrm{atm}}$, Equation (3) becomes:

(4) $$p=p_{0}+\rho g\left(H-z\right)$$

where $p_{0}$ is atmospheric pressure, *z* is the height coordinate with z=0 at the substrate surface, *g* is the gravitational acceleration in the *z* direction, and *H* is the height of the liquid bridge.

Substituting Equation (4) and the curvature Equation (2) into Equation (1), yields the description of the liquid bridge profile as:

(5) $$\frac{R^{\prime \prime }}{{\left(1+R^{\prime 2}\right)}^{3/2}}-\frac{1}{R{\left(1+R^{\prime 2}\right)}^{1/2}}+\frac{\rho g(H-z)}{\sigma }=0$$

The boundary conditions associated with Equation (5) are as follows:

(6) $$\left\{\begin{array}{c} {\left.R\right|}_{z=0}=R_{0}\\ {\left.\frac{dR}{dz}\right|}_{z=0}=\cot\phi_{0}\\ {\left.R\right|}_{z=H}=R_{w} \end{array}\right.$$

where $R_{0}$ is the bottom radius of the liquid bridge, $\phi_{0}$ is the angle between the liquid bridge profile and substrate, and $R_{w}$ is the projection radius of the wire in the z=0 plane. For the studied case, $R_{w}$ reads:

(7) $$R_{w}=R_{c}/\sin\theta$$

where $R_{c}$ is the wire radius, and θ is the angle between the feed wire and the substrate.

Since the solution to Equation (5) cannot be obtained analytically, we proposed Algorithm 1, an iterative approach, to solve it. In the proposed method, $R_{0}$ and $\phi_{0}$ are chosen to be input parameters, to obtain the unknown liquid bridge height, *H*. To calibrate the values of $R_{0}$ and $\phi_{0}$, a high-fidelity numerical model is applied. Since the surface tension coefficient changes with temperature, σ is also calibrated using the numerical model. By combining bisection and quadrature methods, as listed in Algorithm 1, Equation (5) can be solved with these input parameters. It is worth noting, that the proposed method converges quickly when the appropriate input parameters are selected.
**Algorithm 1:** Algorithm to the liquid bridge profile. 
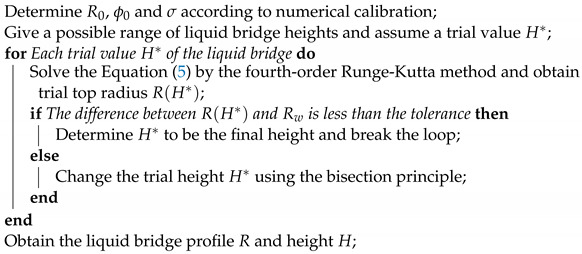


### 2.3. The Transition from Liquid Bridge to Droplet Mode

In the proposed model, above, a liquid bridge is assumed to form between the wire and the substrate. However, as the distance between the wire and the substrate increases, the liquid bridge mode will transition to the droplet mode. Algorithm 1 is used to determine the height of the liquid bridge. The critical condition for the transition to the droplet mode can be expressed as follows.

(8) $$H_{0}⩽H$$

where $H_{0}$ is the initial distance between the wire tip and the substrate. If Equation (8) is not met, the liquid bridge mode may transition to the droplet mode.

**Figure 3:**
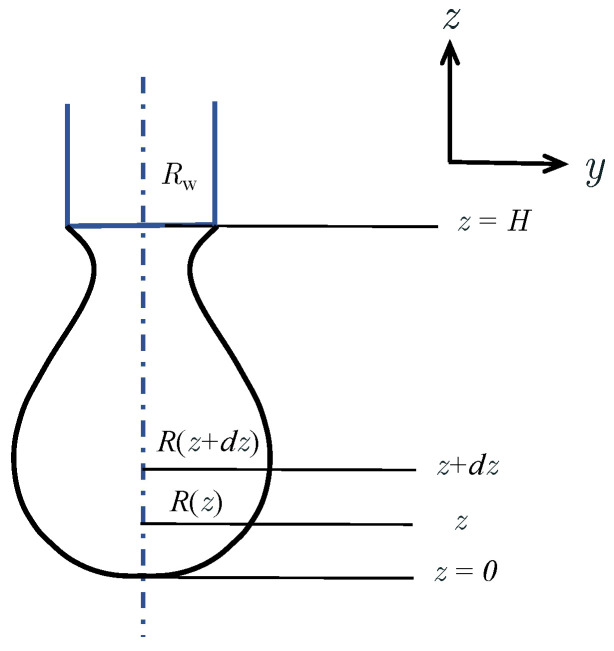
Schematic diagram of the liquid droplet metal transfer mode.

In the droplet mode, the liquid profile is shown in Figure 3, and it is also governed by Equation (5). However, as the liquid becomes a pendant droplet, the boundary conditions change to:

(9) $$\left\{\begin{array}{c} {\left.R\right|}_{z=0}=0\\ {\left.R\right|}_{z=H}=R_{w} \end{array}\right.$$

The bottom tip of the droplet is parabolic, and the two principal curvature radii are equal. So near the tip, one has:

(10) $$z=\frac{1}{2}\frac{R^{2}}{r_{0}}$$

where $r_{0}$ is the tip radius of the droplet.

The profile of the pendant droplet is obtained through a scheme similar to Algorithm 1. To avoid the singularity, Equation (10) is used as the initial value of numerical integration. Once the droplet profile is predicted, the maximum volume of droplet growth can be achieved as:

(11) $$V_{d}=\pi \int_{0}^{H}R^{2}\left(z\right)dz$$

with $V_{d}$ being the maximum volume of each droplet.

Assuming the arc power is sufficient to melt the wire completely, we can calculate the wire melting volume rate ${\dot{V}}_{w}$ as:

(12) $${\dot{V}}_{w}=\pi R_{c}^{2}v_{w}$$

with $v_{w}$ being the wire feed velocity. Therefore, the frequency of the liquid drop is:

(13) $$f=\frac{{\dot{V}}_{w}}{V_{d}}$$

In turn, as the droplet frequency increases, the droplet mode gradually shifts to the liquid bridge mode, resulting in improved quality of formation.

### 2.4. Transition from Liquid Bridge to Wire Stubbing Mode

In the liquid morphology model presented above, it is assumed that the arc power is sufficient to fully melt the wire. However, in practical applications, this is often not the case. If the arc power is too low or the wire feed speed is too fast, it can result in the wire not melting completely and entering the melt pool, which is referred to as wire stubbing mode. In this mode, the tip of the wire collides with the solid part at the bottom of the melt pool, causing significant disruption to the melt pool flow field. As a consequence, irregularities in the solidified track’s morphology can occur. To predict the wire stubbing mode, the following formulations are derived.

**Figure 4:**
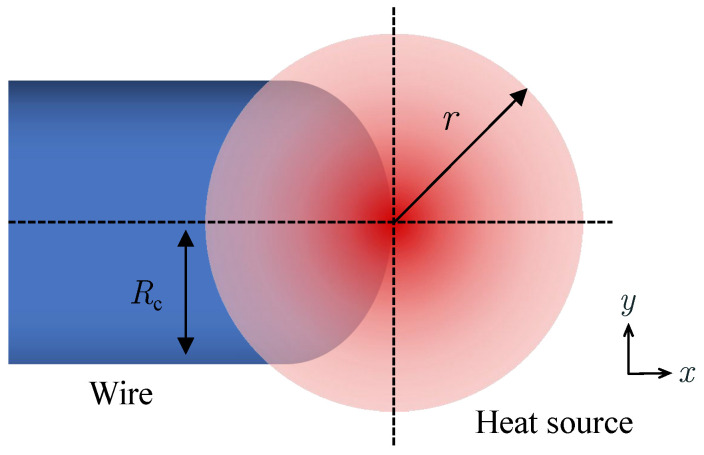
Schematic diagram of interaction area between the heat source and wire.

For the WAAM process, the wire tip and the heat source affected area are illustrated in Figure 4. Despite the complexity of the arc heat source, it can be approximated into a Gaussian surface heat source, based on experimental and numerical investigations [22,23,24]. In this study, the heat source is defined as:

(14) $$q_{\mathrm{input}}=\frac{\eta P_{l}}{\pi r^{2}}\exp\left(-2\frac{x^{2}+y^{2}}{r^{2}}\right)$$

where η is the absorption coefficient, and *r* is the equivalent radius of the heat source, which is a function of current [25,26,27,28]:

(15) $$r=0.533I^{0.2941}$$

with *I* being the current. The heat source power $P_{l}$ is determined as:

(16) $$P_{l}=IU$$

with *U* being the voltage.

The total power absorbed by the wire can be calculated by integrating the affected area:

(17) $$E_{\mathrm{absorb}}=\int \int \;q_{\mathrm{input}}dS=\int \int \;\frac{\eta P_{l}}{\pi r^{2}}\exp\left(-2\frac{x^{2}+y^{2}}{r^{2}}\right)dS$$

Assuming the temperature of the preheated wire to be $T_{p}$, if the wire is fully melted, the temperature at the wire cross-section needs to reach the solidus temperature, $T_{s}$. The power needed for melting satisfies:

(18) $$E_{\mathrm{melt}}=\rho \pi R_{c}^{2}v_{w}c_{p}\left(T_{s}-T_{p}\right)$$

where $c_{p}$ is the specific heat capacity.

According to the conservation of energy, the condition for the complete melting of the wire is:

(19) $$E_{\mathrm{melt}}⩽E_{\mathrm{absorb}}$$

Otherwise, the liquid bridge mode will change to the wire stubbing mode.

### 2.5. High-Fidelity Numerical Model for Model Calibration

In the above model, three input parameters must be calibrated using numerical simulations, including the bottom radius of the liquid bridge $R_{0}$, the angle $\phi_{0}$, and the surface tension coefficient σ, as a function of temperature. The high-fidelity numerical model is set up as follows.

The metal is assumed to be an incompressible mixture in the numerical simulations. The mass conservation equation reads:

(20) ∇·u=0

where u is the fluid velocity. The momentum conservation equation reads:

(21) $$\rho \frac{\partial u}{\partial t}+\rho u\cdot \nabla u=\nabla \cdot \left(\mu \nabla u\right)+\rho g-\nabla p-\rho g\beta \left(T-T_{L}\right)+K_{0}\frac{{\left(1-f_{l}\right)}^{2}}{f_{l}^{3}+B}u+b$$

where μ is the dynamic viscosity, β is the fluid thermal expansion coefficient, and $T_{L}$ is the liquidus temperature. $K_{0}$ is the Carman–Kozeny coefficient of the mushy zone flowing through a porous media [29], $f_{l}$ is the liquid volume fraction, *B* is a small constant to avoid the singularity, and b is the body force. The momentum equation considers the buoyancy force under Boussinesq’s assumption and Darcy’s damping effect.

At the gas–liquid interface, the surface tension and Marangoni forces are defined as follows:

(22) $$f_{\mathrm{surface}}=\sigma n\kappa +\frac{d\sigma }{dT}\left[\nabla T-n\left(n\cdot \nabla T\right)\right]$$

where n is the normal vector of the surface, and $\frac{d\sigma }{dT}$ is the of surface tension gradient.

The arc pressure at the gas–liquid interface is assumed to follow a Gaussian distribution, as follows [27]:

(23) $$p_{\mathrm{arc}}=\frac{\mu_{m}I^{2}}{8\pi \sigma_{a}^{2}}\exp\left(-\frac{x^{2}+y^{2}}{2\sigma_{a}^{2}}\right)$$

where $\mu_{m}$ is the magnetic permeability, and $\sigma_{a}$ is the Gaussian pressure parameter.

The recoil pressure caused by metal evaporation is as follows:

(24) $$p_{\mathrm{recoil}}=0.54p_{0}\exp\left[\frac{L_{v}M\left(T-T_{v}\right)}{R_{g}TT_{v}}\right]$$

where $L_{v}$ is the latent heat of vaporization, *M* is the molar mass, and $R_{g}$ is the universal gas constant. The surface tension, arc pressure, and recoil pressure are converted into body force b, by using the volume fraction.

The free surface evolution is solved by the volume of fluid (VOF) method, as follows:

(25) $$\frac{\partial f_{m}}{\partial t}+\left(u\cdot \nabla \right)f_{m}=0$$

where $f_{m}$ is the volume fraction of the metal.

The energy conservation equation reads:

(26) $$\frac{\partial }{\partial t}\left(\rho h\right)+u\cdot \nabla \left(\rho h\right)=\nabla \cdot \left(k\nabla T\right)-\frac{\partial }{\partial t}\left(\rho \Delta h\right)-u\cdot \nabla \left(\rho \Delta h\right)+Q$$

where *h* is the enthalpy, *k* is the heat conductivity, Δh is the latent enthalpy change of the alloy, and *Q* includes the volume heat source and losses. The arc heat source is given in Equation (14). The heat losses are defined as follows.

(27) $$q_{\mathrm{loss}\;}=\varepsilon \sigma_{b}\left(T^{4}-T_{a}^{4}\right)+h_{c}\left(T-T_{a}\right)+\frac{0.82L_{v}M}{\sqrt{2\pi MR_{g}T}}p_{0}\exp\left[\frac{L_{v}M\left(T-T_{v}\right)}{R_{g}TT_{v}}\right]$$

The right-hand side of Equation (27) includes the radiation, convection, and vaporization heat losses. In Equation (27), ε is the emissivity, $\sigma_{b}$ is the Stefan–Boltzmann constant, $T_{a}$ is the ambient temperature, and $h_{c}$ is the convection coefficient.

**Figure A1:**
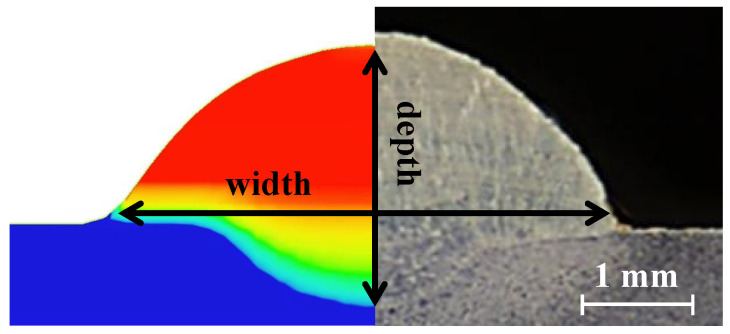
Transverse cross-sectional view of the molten track, where the left part is from the numerical results and the right part from the experiment [38].

The finite volume method (FVM) is used to solve the model. The momentum equation is solved using an operator-splitting scheme [30]. A second-order upwind scheme is used for the convection term [31], and the first-order Euler method is applied for time integration [32]. The validation of the high-fidelity numerical simulation model is detailed in Appendix A (see Figure A1). The numerical results are in good agreement with the experimental data. Once the numerical results are obtained, they can calibrate the bottom radius $R_{0}$, the angle $\phi_{0}$, and the surface tension coefficient σ.

### 2.6. Procedures of the Prediction Model

For the proposed fast prediction model, the procedures to predict the liquid metal transfer mode are as follows.

1. Calibrate the bottom radius $R_{0}$, the angle $\phi_{0}$, and the surface tension coefficient σ, by high-fidelity numerical simulations.
2. Calculate the liquid bridge profile and height *H*, using Equation (5).
3. If Equation (8) is unsatisfied, the initial distance between the wire and the substrate exceeds the liquid bridge height limit, and the liquid bridge will develop into the droplet transfer mode. Calculate the dripping frequency of droplets using Equation (13).
4. If the energy conservation condition in Equation (19) is not satisfied, the liquid bridge will develop into the wire stubbing mode.

## 3. Results and Discussion

### 3.1. Model Calibration by High-Fidelity Numerical Simulations

**Figure 5:**
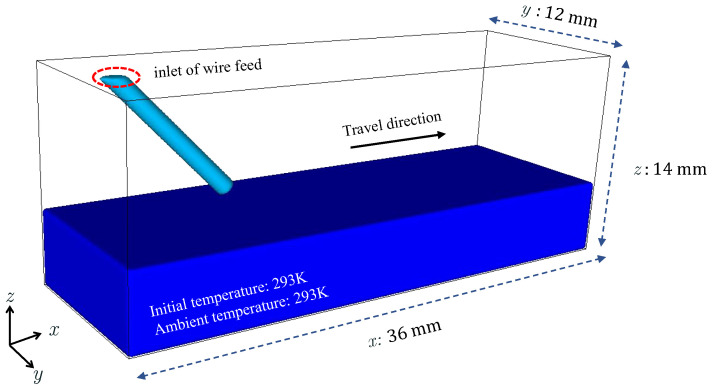
High-fidelity numerical model used for calibration of the prediction model.

Before using the proposed model to predict the liquid metal transfer mode, the numerical examples shown in Figure 5 are conducted, to calibrate the prediction model.

In this case, *x*, *y*, and *z* are the travel, the transverse, and the height directions, respectively. The size of the calculation domain is 3.6 cm × 1.2 cm × 1.4 cm. The material used for both substrate and wire is mild steel, with its material parameters [33,34] listed in Table 1 and Table 2. The thermal conductivity and specific heat capacity are temperature-dependent and are linearly interpolated from the values listed in Table 2, while other parameters remain constant.

Three cases are conducted, with a constant travel speed of 1 cm/s, and wire feed speeds of 2 cm/s, 3 cm/s, and 4 cm/s, respectively. The selected wire feed speeds are in the reasonable range of experimental conditions for WAAM. The arc heat source power is 1440 W and the wire diameter is 1.2 mm. The cell size is 0.01 cm, leading to 6,048,000 cells. The temperature distribution, with respect to the *z* coordinate on the liquid bridge, is fitted according to an average of the results of the three examples. The second-order Gaussian function is used to fit the curve, and the coefficient of determination (denoting the goodness of fit), is 0.99. The fitting results are as follows.

(28) $$T\left(z\right)=a_{1}e^{-{\left[\left(z-b_{1}\right)/c_{1}\right]}^{2}}+a_{2}e^{-{\left[\left(z-b_{2}\right)/c_{2}\right]}^{2}}$$

where $a_{1}=546$, $b_{1}=-0.0837$, $c_{1}=0.01505$, $a_{2}=1882$, $b_{2}=-0.1095$, and $c_{2}=0.8174$. Since a suitable dataset for temperature-dependent surface tension coefficients of the liquid metal is not currently available, a linear fitting is used to obtain the surface tension coefficient:

(29) $$\sigma =\sigma_{0}+\frac{d\sigma }{dT}\left[T\left(z\right)-T_{L}\right]$$

where $\sigma_{0}$ is the surface tension coefficient at the liquidus temperature $T_{L}$, and $\frac{d\sigma }{dT}$ is given as a constant [34] in Table 1. By substituting Equation (28) into Equation (29), the surface tension used in Equation (5) is calibrated.

Additional cases are required to calibrate the bottom radius $R_{0}$ and angle $\phi_{0}$ as input parameters in the prediction model. This is because, unlike the surface tension coefficient, which is not very sensitive to the process parameters, $R_{0}$ depends on the wire feed speed and travel speed. Therefore, nine cases were carried out to calibrate the two parameters. The setups for these nine cases are listed in Table 3, while others are the same as the above case.

The $R_{0}$ in Table 3 was obtained from each numerical case by taking the average over a period of time. Ten different time instances were selected, and the statistical results were regressed. Here, parameter ξ is introduced, which is defined as the ratio of the wire feed speed to travel speed. A regression relationship between ξ and $R_{0}$ can be established:

(30) $$R\left(\xi \right)=a\xi^{2}+b\xi +c$$

where the parameters *a*, *b*, and *c* are 2.3 × 10${}^{-3}$, 2.5 × 10${}^{-2}$, and 5.5 × 10${}^{-2}$, respectively. Equation (30) is then used to calibrate the liquid bridge bottom radius in the liquid bridge profile, Equation (5).

The same approach is used for the calibration of angle $\phi_{0}$, by averaging the numerical results at various time instances. However, from the results of the high-fidelity numerical model, it is found that $\phi_{0}$ is not sensitive to the values taken for the wire feed speed and travel speed. In the given range of parameters, the average of $\phi_{0}$ is around 10.1 degrees, with no significant deviation, and thus is chosen as the input of Equation (5). Considering that the predictions in the two cases are insufficient to illustrate the model’s prediction capability, more predictions are shown in Section 3.4, in the process parameter window investigation.

### 3.2. Liquid Bridge Profile and Critical Height Validation

Two cases are presented to validate the profile predicted by the proposed model. In the first case, the wire feed speed is 2 cm/s, and the calibrated bottom radius $R_{0}$ is 0.12 cm. In the second case, the wire feed speed is 1.34 cm/s, and the calibrated bottom radius $R_{0}$ is 0.10 cm. Other setups are the same as the numerical cases used in Section 3.1.

**Figure 6:**
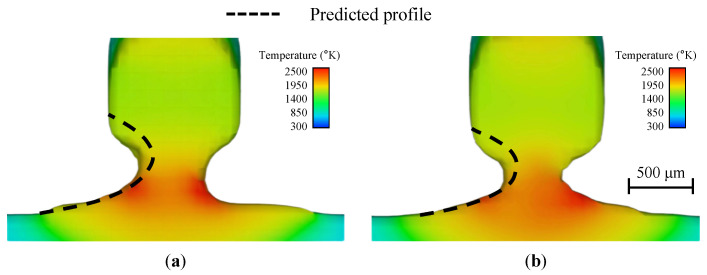
Comparison of the predicted profile (dashed line) and the numerical results for (**a**) wire feed speed of 2 cm/s, (**b**) wire feed speed of 1.34 cm/s.

The high-fidelity numerical and proposed prediction models’ results are presented in Figure 6. It is demonstrated that the predicted profiles are similar to the results obtained by the high-fidelity numerical model. The profile of the neck region of the liquid bridge deviates slightly compared to the numerical results. This deviation may be because the proposed prediction model neglects some surface forces acting on the fluid. However, the overall profile and bridge height results agree well with the numerical results. For the liquid bridge heights, the height obtained by the prediction model is 0.10 cm in the first case, while the height calculated by the high-fidelity numerical simulation is 0.09 cm and the relative error is 11.1%. In the second case, the height of the liquid bridge calculated by the proposed model is 0.085 cm, while the height achieved by the numerical model is 0.08 cm and the relative error is 6.3%.

**Figure 7:**
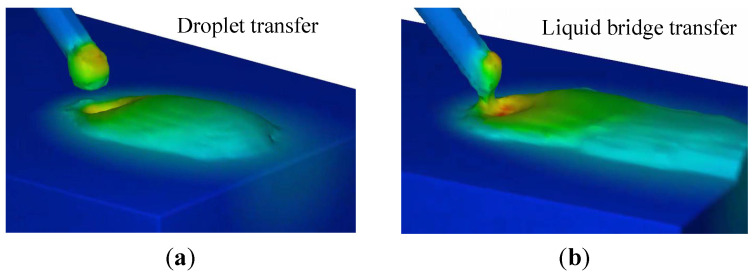
Verification of critical condition for the transition from liquid bridge to droplet mode: (**a**) initial distance $H_{0}=0.14$ cm, (**b**) initial distance $H_{0}=0.12$ cm.

For the predicted liquid bridge profiles, the critical condition for the occurrence of the droplet mode can be determined. According to Equation (8), if the distance between the substrate and wire tip, $H_{0}$, exceeds the liquid bridge height *H*, the liquid bridge changes to the droplet mode. Two cases are presented here, to verify the proposed model’s capability in predicting the liquid bridge’s critical height. In the first case, the initial distance between the substrate and wire tip, $H_{0}$, is 0.12 cm. In the second case, $H_{0}$ is 0.14 cm. The wire feed speed is 4 cm/s in both cases. The numerical results are shown in Figure 7.

The numerical results suggest that the first case is droplet mode, and the second is liquid bridge mode. From the proposed model, the critical height *H*, is 0.12 cm, and the two cases should be droplet and liquid bridge modes, respectively, according to Equation (8). A stable liquid bridge cannot be formed when the distance between the wire and the substrate exceeds the predicted maximum liquid bridge height. It is demonstrated that the predicted results agree well with the high-fidelity numerical simulations.

### 3.3. Droplet Profile and Frequency Validation

The proposed model can be used to predict the droplet profile and dripping frequency for the droplet mode. Therefore, two cases are presented here to verify the droplet profile and dripping frequency.

**Figure 8:**
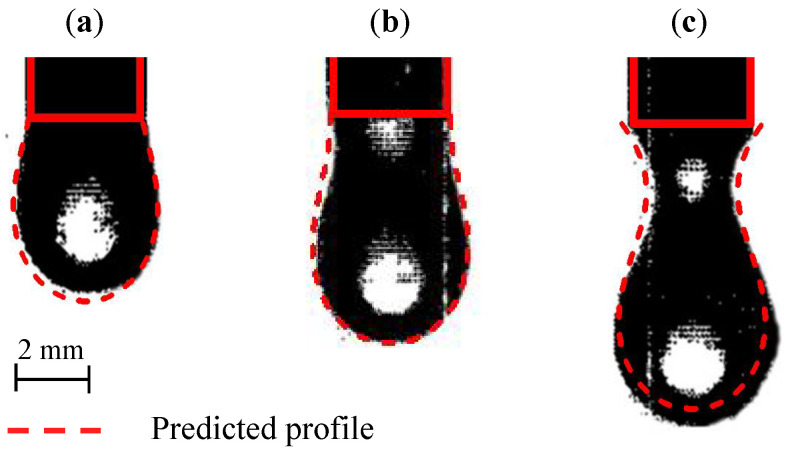
Droplet profile predictions. The pictures in black and white are from experiments [35], and the red dashed lines are predicted results: (**a**) t=2092 ms, (**b**) t=2990 ms, (**c**) t=3075 ms.

The first case is the dripping of water droplets. The predicted results have been compared to the experimental profiles by Zhang et al. [35]. In the water droplet experiment, the water density is 1000 kg/m${}^{3}$, the surface tension coefficient is 0.0728 N/m, the outer radius of the tube is 0.16 cm, and the tube flow rate is 1 mL/min. The profiles of droplets at different time instances are shown in Figure 8, where the predicted profiles obtained by Equation (5) are in good agreement with the experimental data [35]. The comparison shown in Figure 8, demonstrates that the proposed model can predict the droplet profiles accurately at different time instances.

**Figure 9:**
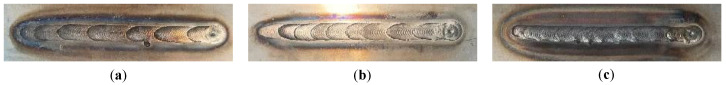
WAAM experiment for dripping frequency under different wire feed speeds: (**a**) wire feed speed of 0.5 cm/s, (**b**) wire feed speed of 0.67 cm/s, (**c**) wire feed speed of 0.83 cm/s.

As detailed in Appendix B, we conducted WAAM experiments to investigate the dripping frequency. The experiments were conducted using the process parameters listed in Table A2, with a duration of 1 min. The results obtained are plotted in Figure 9, which reveals that all three experimental cases exhibited droplet modes. In this mode, the liquid metal droplet grows gradually as the wire is fed. However, when the surface tension is insufficient to maintain the droplet shape, it separates from the wire, causing the periodic impact of these droplets to yield ripples on the solidified specimen. Since the droplets cannot be observed directly during the experiment, their number and dripping frequency are calculated indirectly, based on the spacing of the ripples on the solidified specimen. A comparison between the experimental and theoretical results is presented in Table 4. The experimental findings indicate that both the number of droplets and dripping frequency increase with the wire feed speed, which is consistent with Equation (13). The predictions are in good agreement with the experimental trend, although the number of droplets and dripping frequency from the experiment surpasses the predicted values. The relative error of the dripping frequency ranges from 10.2% to 18.9%, which may be attributed to unstable conditions during the experiment, such as arc pressure oscillation and equipment vibration. Additionally, the neglected electromagnetic forces and pinch effects in the prediction model could have accelerated the dripping frequency of droplets. Despite these discrepancies, the proposed model still fulfills the process parameter control requirements to a certain extent.

### 3.4. Process Parameter Window Considering Liquid Bridge–Droplet Transition

In the WAAM process, the liquid bridge mode is preferred, but identifying the critical conditions for the transition between the liquid bridge and droplet modes through experiments can be expensive. High-fidelity numerical simulations are also computationally intensive. For example, the computational time of a typical numerical case established in this study is approximately 24 h, using a workstation with 40 cores CPU (Intel Xeon 6248). Not to mention, that at least dozens of such examples are required to obtain a process parameter window. Therefore, the proposed fast prediction model for obtaining a process parameter window is crucial.

**Figure 10:**
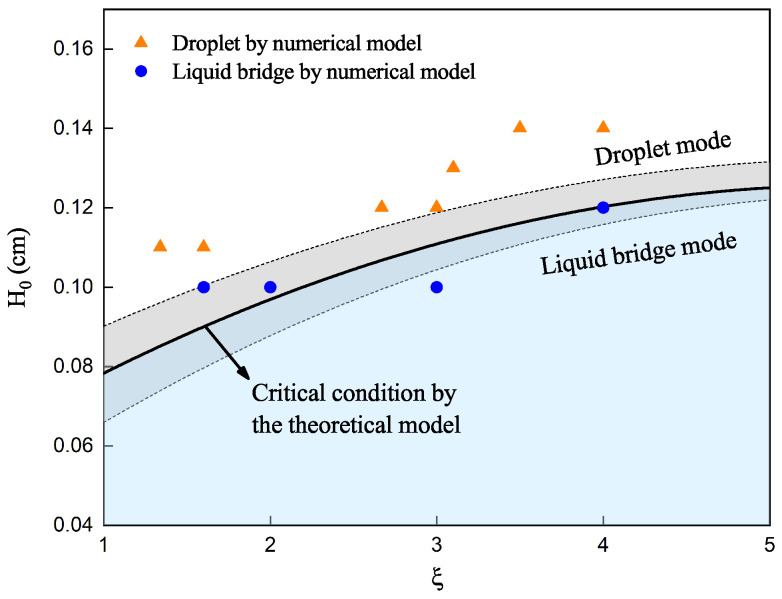
Process parameter window considering liquid bridge–droplet transition.

With the proposed prediction model, it is possible to determine the critical condition for the droplet mode, as shown in Equation (8). This model allows for the presentation of a process parameter window that considers the liquid bridge–droplet transition, with a focus on two key process parameters: the initial distance between the substrate and wire tip ($H_{0}$) and the wire feed speed to travel speed ratio (ξ). The process parameter window generated by the proposed model is illustrated in Figure 10. The process parameters can be clearly divided into two zones in the figure: the upper area represents the droplet mode zone, and the lower area represents the liquid bridge mode zone. The black line is obtained using Equation (5), while the shadowed error bar zone is obtained by selecting different calibrated $R_{0}$ values, taking into account the standard deviation in Table 3.

**Figure 11:**
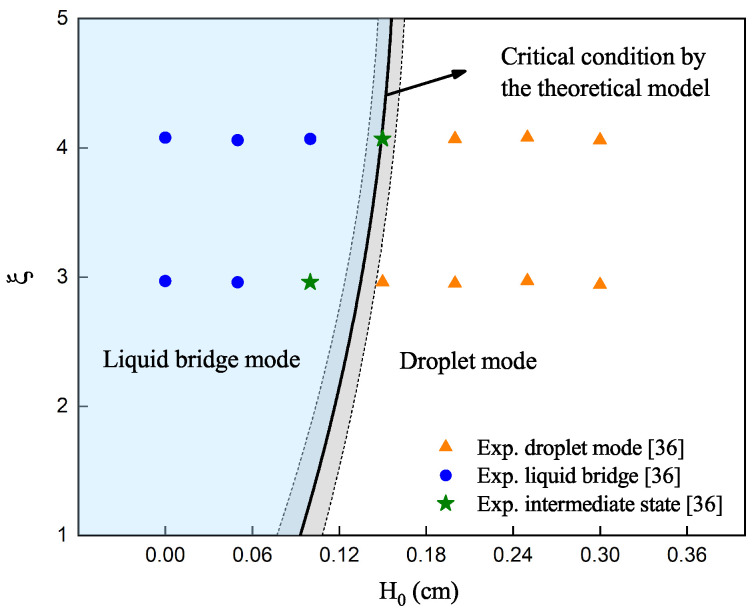
Liquid bridge and droplet modes predicted by the proposed model and observed from experiments [36].

A total of eleven high-fidelity numerical cases were conducted, to verify the accuracy of the proposed prediction model, with their setups summarized in Table 5. Figure 10 presents a comparison of the numerical simulation results and the predictions of the proposed model, where the triangles and circles represent the droplet and liquid bridge modes, respectively. From the comparison, one can find that the predictions from the proposed model are in good agreement with their counterparts from the numerical simulations. However, the proposed model takes only a few minutes to complete the prediction, while high-fidelity numerical simulations often require a couple of days. The proposed model is also compared with experiments conducted by Wang et al. [36]. Fourteen experimental results, using wires with a diameter of 2.4 mm, were chosen for comparison. In these experiments, the distance $H_{0}$, between the substrate and wire, ranged from 0–3 mm, and the wire feed speeds were 0.8 m/min and 1.1 m/min. Details of the setups can be found in Figure 17 in [36]. Figure 11 presents a comparison between the experimental results and the predictions of the proposed model, with triangles, circles, and stars representing the droplet, liquid bridge, and intermediate states observed from experiments, respectively. The predictions are in good agreement with experiments. It is demonstrated that the proposed model can efficiently predict the critical conditions of the liquid bridge–droplet transition and provide guidance for practical engineering applications.

### 3.5. Process Parameter Window Considering Liquid Bridge–Wire Stubbing Transition

In the WAAM process, the wire stubbing mode must be avoided as much as possible, because it can lead to poor forming quality. The proposed prediction model can be used to determine the critical conditions for the wire stubbing mode, as shown in Equation (19).

**Figure 12:**
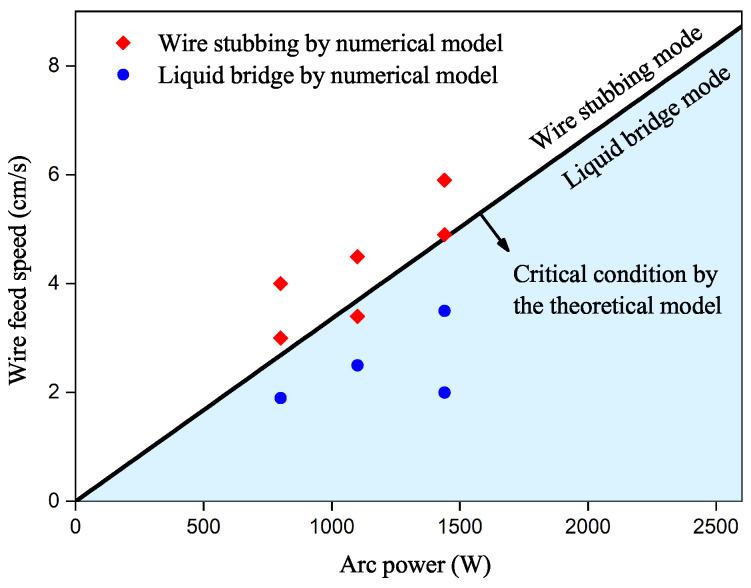
Process parameter window considering liquid bridge–wire stubbing transition.

Using the proposed model, we present a process parameter window that considers the transition of the liquid bridge–wire stubbing. The two most critical parameters, i.e., the arc power and the wire feed speed, are considered. The wire feed speed ranges from 0 to 9 cm/s, while the arc power ranges from 0 to 2600 W. The process parameter window depicted by the proposed model is shown in Figure 12. The figure clearly divides the process parameters into two zones. The top left zone corresponds to the liquid bridge mode, while the bottom right zone is for the wire stubbing mode.

Ten high-fidelity numerical cases were conducted, to validate the accuracy of the prediction model. The material parameters and setups used in these cases are listed in Table 1 and Table 6, respectively. Judging whether the numerical results predict the liquid bridge or wire stubbing mode in all ten examples is challenging, as it cannot be directly observed from the high-fidelity numerical simulations. Therefore, the deposition efficiency is defined, and proposed to make such judgments. This parameter represents the ratio of the actual volume increase in the specimen after solidification, to the volume rate of the wire feed, and an ideal deposition efficiency should be 100%. If the wire is not completely melted before it enters the melt pool in the numerical simulations, the deposition efficiency will be less than 100%, indicating the occurrence of wire stubbing mode. In this study, a 90% deposition rate is used as a critical condition to account for any numerical errors. All ten cases are illustrated in Figure 12, with diamonds representing wire stubbing mode and circles denoting liquid bridge mode. The proposed prediction model is shown to agree well with the numerical results, effectively predicting the critical conditions of the liquid bridge–wire stubbing transition; and so could be used to guide engineering practices. It is identified that the wire stubbing mode is less likely to occur with higher arc power and lower wire feed speed.

## 4. Conclusions

In this study, a fast prediction model for identifying liquid metal transfer modes in wire arc additive manufacturing is proposed. By utilizing the Young–Laplace equation, momentum equation, and energy conservation, the profile and height of the favorable liquid bridge are obtained, which is crucial in determining the critical conditions for transitioning between different modes. Specifically, the critical height of the liquid bridge is used to determine the liquid bridge–droplet transition, while the energy conservation principle is applied to derive the critical condition for the liquid bridge–wire stubbing transition. In addition, the dripping frequency of the liquid drop can be predicted. High-fidelity numerical simulations are used to calibrate the surface tension coefficient, the bottom radius of the liquid bridge, and the angle between the liquid bridge profile and substrate, to close the solution of the proposed model. Numerical cases and experiments were carried out to validate the accuracy of the prediction model, and the results show that the error ranges from 10.2% to 18.9% for the dripping frequency in the droplet mode, and from 6.3% to 11.1% for the liquid bridge height in the liquid bridge mode. Moreover, it is demonstrated that the proposed model can accurately predict the transition from liquid bridge to droplet and wire stubbing modes.

Based on the proposed prediction model, a process parameter window, considering the transition from liquid bridge mode to droplet and wire stubbing modes, is presented. The model is demonstrated to be effective and efficient in optimizing the process parameters. In the WAAM process, the choice of process parameters and the liquid metal transfer modes can have significant impacts on the quality of the final build. Therefore, it is of far-reaching significance to establish an efficient prediction model and corresponding process parameter windows in actual production. The proposed model, therefore, has practical significance in guiding the actual industrial production process and improving the fabrication quality.

In addition, the proposed model in this study has some limitations that should be acknowledged. The model assumes a steady-state liquid transport process, and considers most material properties to be constants. Potential improvements include, using temperature-dependent coefficients and considering the thermocapillary stress, e.g., the semi-analytical model for the apparent slip length of Poiseuille and Couette flows [37]. The influence of electromagnetic forces, the pinch effect, and wire melting rate considering different power sources, are also neglected. Future research could incorporate these factors into the model to enhance its accuracy and applicability.

## Figures and Tables

**Table 1:** Material properties of the mild steel.

Material Properties	Value	Units
Density, ρ	7200	kg m${}^{-3}$
Ambient pressure, $p_{0}$	0.1	MPa
Solidus temperature, $T_{s}$	1750	K
Liquidus temperature, $T_{L}$	1800	K
Vaporization temperature, $T_{v}$	3143	K
Viscosity, μ	6 × 10${}^{-3}$	Pa · s
Thermal conductivity, *k*	27 (293 K)	W m${}^{-1}$ K${}^{-1}$
Specific heat capacity, $c_{p}$	710 (293 K)	J kg${}^{-1}$ K${}^{-1}$
Latent heat of fusion, $L_{m}$	2.47 × 10${}^{5}$	J kg${}^{-1}$
Latent heat of vaporization, $L_{v}$	6.08 × 10${}^{6}$	J kg${}^{-1}$
Molar mass, *M*	5.2 × 10${}^{-3}$	kg mol${}^{-1}$
Surface tension coefficient, $\sigma_{0}$	1.2	N m${}^{-1}$
Surface tension gradient, $\frac{d\sigma }{dT}$	−1 × 10${}^{-4}$	N m${}^{-1}$ K${}^{-1}$
Convection coefficient, $h_{c}$	10	W m${}^{-2}$ K${}^{-1}$

**Table 2:** Temperature dependent properties of the mild steel.

Temperature (K)	293	800	1300	1800
Thermal conductivity				
(W m${}^{-1}$ K${}^{-1}$)	27.0	35.4	43.7	52.0
Specific heat capacity				
(J kg${}^{-1}$ K${}^{-1}$)	710.0	740.2	770.1	800.0

**Table 3:** Numerical cases for calibrating the bottom radius.

Case	Wire Feed Speed (cm/s)	Travel Speed (cm/s)	ξ	Bottom Radius $R_{0}$ (cm)	Standard Deviation (cm)
1	0.50	0.75	0.67	0.085	0.0195
2	0.50	0.50	1.00	0.110	0.0241
3	0.75	0.50	1.50	0.116	0.0231
4	1.80	1.00	1.80	0.137	0.0245
5	2.20	1.00	2.20	0.143	0.0160
6	2.00	0.75	2.67	0.149	0.0191
7	4.00	1.25	3.00	0.170	0.0125
8	2.00	0.5	4.00	0.177	0.0137
9	2.25	0.5	4.50	0.206	0.0191

**Table 4:** Comparison of droplet number and dripping frequency between experimental data and theoretical results.

	Case 1	Case 2	Case 3
Wire feed speed	0.50 cm/s	0.67 cm/s	0.83 cm/s
Experimental number of droplets	7	10	13
Theoretical number of droplets (taking floor)	6	8	10
Experimental dripping frequency	0.117 Hz	0.167 Hz	0.217 Hz
Theoretical dripping frequency	0.105 Hz	0.141 Hz	0.176 Hz
Relative error (for frequency)	10.2%	15.6%	18.9%

**Table 5:** Numerical cases to validate the prediction model for liquid bridge–droplet transition.

Case	Wire Feed Speed (cm/s)	Travel Speed (cm/s)	$H_{0}$ (cm)	Power (W)
1	2.00	1.25	0.10	1440
2	1.00	0.50	0.10	1440
3	1.50	0.50	0.10	1440
4	2.00	1.25	0.11	1440
5	1.00	0.75	0.11	1440
6	4.00	1.50	0.12	1440
7	1.50	0.50	0.12	1440
8	4.00	1.00	0.12	1440
9	2.20	0.70	0.13	1440
10	3.50	1.00	0.14	1440
11	4.00	1.00	0.14	1440

**Table 6:** Numerical cases to validate the prediction model for the liquid bridge–wire stubbing mode transition.

Case	Wire Feed Speed (cm/s)	Travel Speed (cm/s)	Power (W)
1	2.00	1.00	1440
2	4.00	1.00	1440
3	5.00	1.00	1440
4	6.00	1.00	1440
5	2.50	1.00	1100
6	3.50	1.00	1100
7	4.50	1.00	1100
8	2.00	1.00	800
9	3.00	1.00	800
10	4.00	1.00	800

**Figure A2:**
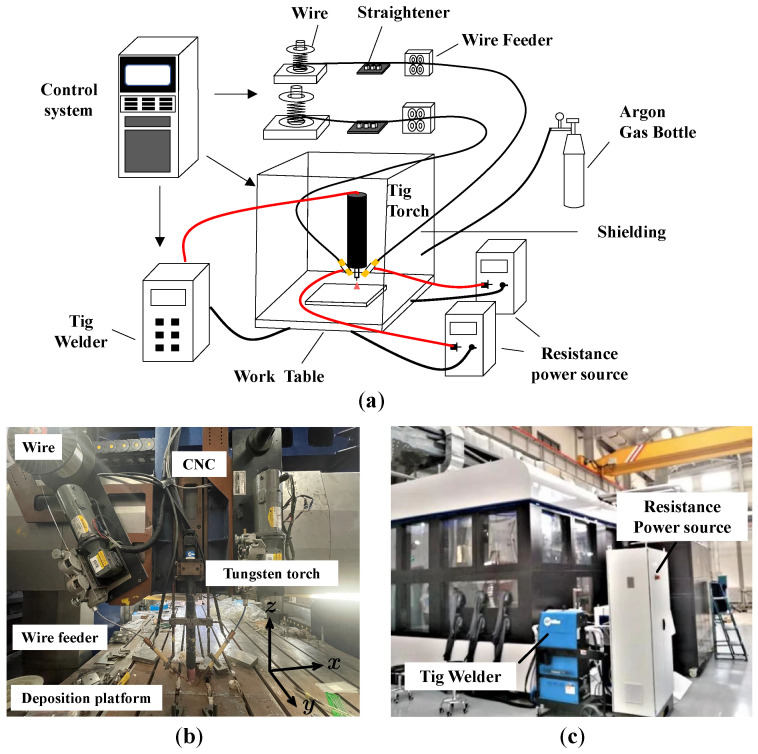
The WAAM equipment: (**a**) schematic diagram of WAAM equipment, (**b**) equipment details, (**c**) photo of the equipment.

## Data Availability

The main data supporting the work are available within the article. Extra data are available from the corresponding author upon reasonable request.

## Appendix A. Numerical Model Validation

The experiment conducted by Ogino et al. [38] was addressed, using the numerical model for its validation. The material properties used in this numerical model are summarized in Table 1, and the process parameters are set to wire feed speed of 5 cm/s, travel speed of 1 cm/s, and heat source power of 1440 W. The comparison of the deposition layer’s size and shape between the numerical simulation and experimental results is shown in Figure A1, indicating the accuracy of the numerical model. On the transverse cross-section, the average bead width and depth from the experiment are 0.46 cm and 0.26 cm, respectively. The bead width and depth from the numerical simulation are 0.49 cm and 0.24 cm, respectively. The average width and depth errors are found to be 6.5% and 7.6%, respectively, indicating that the morphology and dimensions of the bead from the numerical results agree well with their counterparts from the experimental data.

**Table A1:** Comparison of melt pool width and depth between experiment data and simulation results.

	Width	Depth
Experimental	0.46 cm	0.26 cm
Numerical simulation	0.49 cm	0.24 cm
Relative error	6.5%	7.6%

## Appendix B. Experimental Equipment

The WAAM equipment utilized in this study is shown in Figure A2a. The equipment comprises a control system, a computer numerical control (CNC) machine tool, an arc power supply machine, a tungsten inert gas torch installed on the CNC machine tool, two hot-wire resistance power sources, and two wire feeders. As depicted in Figure A2b, the wire feed tubes are situated on both sides of the torch at a 45-degree oblique downward angle, and two wire feeders manage the feed speed. The travel speed of the CNC machine tools is regulated by the motion programs written in the control system.

Regarding the power source, a direct current power source is used, with a current of *I* = 95 A and a voltage of U=15.16 ± 0.6 V. The current is obtained by averaging the square wave current over time, and the voltage is measured at the tungsten inert gas torch. The polarity of the power supply is positive for the torch, and zero potential for the substrate. The power of the arc heat source is calculated by multiplying the current by the voltage.

The wire is fed to the bottom of the welding torch, heated by the heat source, melted into liquid metal droplets, and deposited on the substrate. The scanning direction is along the *x* direction. With the movement of the machine tool, the designed track is deposited to complete the WAAM manufacturing process.

**Table A2:** Process parameters used in the experiments.

Parameter	Value (Unit)
Arc power	1440 W
Wire diameter	1.2 mm
Wire feed speed	0.5–0.83 cm/s
Travel speed	0.167 cm/s
